# Development of Highly Informative Genome-Wide Single Sequence Repeat Markers for Breeding Applications in Sesame and Construction of a Web Resource: SisatBase

**DOI:** 10.3389/fpls.2017.01470

**Published:** 2017-08-22

**Authors:** Komivi Dossa, Jingyin Yu, Boshou Liao, Ndiaga Cisse, Xiurong Zhang

**Affiliations:** ^1^Key Laboratory of Biology and Genetic Improvement of Oil Crops, Oil Crops Research Institute of the Chinese Academy of Agricultural Sciences, Ministry of Agriculture Wuhan, China; ^2^Centre d’Etudes Régional pour l’Amélioration de l’Adaptation à la Sécheresse Thiès, Senegal

**Keywords:** sesame, microsatellite, web resource, informative markers, molecular breeding

## Abstract

The sequencing of the full nuclear genome of sesame (*Sesamum indicum* L.) provides the platform for functional analyses of genome components and their application in breeding programs. Although the importance of microsatellites markers or simple sequence repeats (SSR) in crop genotyping, genetics, and breeding applications is well established, only a little information exist concerning SSRs at the whole genome level in sesame. In addition, SSRs represent a suitable marker type for sesame molecular breeding in developing countries where it is mainly grown. In this study, we identified 138,194 genome-wide SSRs of which 76.5% were physically mapped onto the 13 pseudo-chromosomes. Among these SSRs, up to three primers pairs were supplied for 101,930 SSRs and used to *in silico* amplify the reference genome together with two newly sequenced sesame accessions. A total of 79,957 SSRs (78%) were polymorphic between the three genomes thereby suggesting their promising use in different genomics-assisted breeding applications. From these polymorphic SSRs, 23 were selected and validated to have high polymorphic potential in 48 sesame accessions from different growing areas of Africa. Furthermore, we have developed an online user-friendly database, SisatBase (http://www.sesame-bioinfo.org/SisatBase/), which provides free access to SSRs data as well as an integrated platform for functional analyses. Altogether, the reference SSR and SisatBase would serve as useful resources for genetic assessment, genomic studies, and breeding advancement in sesame, especially in developing countries.

## Introduction

During the past years, the development in genetic studies and decrease of genotyping costs, have resulted in the rapid growth of the use of molecular markers (Kantartzi, 2013). Different genetic marker systems have been developed including restriction fragment length polymorphism (RFLP), randomly amplified polymorphic DNA (RAPD), amplified fragment length polymorphism (AFLP), sequence-related amplified polymorphism (SRAP), Diversity Arrays Technology (DArT), restriction-site associated DNA sequencing (RADseq), single-nucleotide polymorphism (SNP), specific-locus amplified fragment sequencing (SLAFseq), and random selective amplification of microsatellite polymorphic loci (RSAMPL). However, simple sequence repeats (SSR) also known as microsatellite has become the molecular marker of choice because of its versatility, operational flexibility, and low-cost. This has provided the foundation for its successful application in a wide range of fundamental and applicable fields, such as, genetic diversity, linkage/association mapping of gene/QTL, marker-assisted selection (MAS), variety identification, and evolution analysis (Jiao et al., 2012; Zhang Q. et al., 2012; Li et al., 2014; Shi et al., 2014; Dossa et al., 2016c).

SSRs are relatively short tandem repeats (STRs) of DNA that are widely distributed throughout whole genomic sequences (Sharma, 2007). They are present in coding regions but are more abundant in non-coding regions (Hancock, 1995). They are characterized by a high co-dominant inheritance, reproducibility, and multi-allelic variation (Morgante and Olivieri, 1993; Kalia et al., 2011). In addition, SSRs have been demonstrated to have several important biological functions including the regulation of chromatin organization, DNA metabolic processes, gene activity, and RNA structure (Li et al., 2002, 2004).

Sesame (*Sesamum indicum* L.) is an emerging oil crop in the world with one of the highest oil content (up to 64%) and quality (Dossa et al., 2017) among major oilseed crops. It is mainly grown in developing countries, as such, its improvement through modern molecular breeding techniques has lagged behind other oilseed crops. Up to now, different types of molecular markers have been developed and applied to sesame genotyping and breeding efforts, such as RAPD (Bhat et al., 1999; Ercan et al., 2004), inter-SSR (ISSR) (Kim et al., 2002), AFLP (Laurentin and Karlovsky, 2006), but SSR has been the preferential marker (Zhang H. et al., 2012; Zhang Y. et al., 2012; Yepuri et al., 2013; Wei et al., 2014; Dossa et al., 2016c). Although their importance in gene mapping and MAS, only few SSR markers are available for sesame research and the available ones fail to adequately represent the entire genome (Dossa, 2016). More importantly, there is no database to search for sesame SSR information at the whole genome level and to perform functional analyses, as developed in other crops such as chickpea (CicArMiSatDB: Doddamani et al., 2014), (CMsDB: Parida et al., 2015), *Cucumis melo* (CmMDb: Bhawna et al., 2015), tomato (TomSatDB: Iquebal et al., 2013), sugar beet (SBMDb: Iquebal et al., 2015), brassicas (Shi et al., 2014), etc.

The completion of the full nuclear genome sequence (Wang et al., 2014a) recently updated (Wang et al., 2016) and the newly sequenced landraces (Wei et al., 2015, 2016) provide a cardinal framework to identify highly informative SSRs at the whole genome level. In this study, we took advantage of these three genome sequence resources and provided not only a large amount of genome-wide informative SSR markers for large-scale genotyping and breeding research in sesame, but also a user-friendly online database for convenient search and functional analyses of SSRs.

## Materials and Methods

### Data Source

Three genome sequences of the cultivated sesame including the reference genome from the elite variety “Zhongzhi13” (Wang et al., 2014a, 2016) and the genome sequences of the landraces “Baizhima” and “Mishuozhima” (Wei et al., 2015, 2016) were downloaded from Sinbase^1^ (Wang et al., 2014b) and SesameFG^2^ (Wei et al., 2017), respectively. It is noteworthy that in this study, the latest version (v2) of the reference genome (Wang et al., 2016) with 13 pseudo-chromosomes (309 Mb) was employed for identifying microsatellites while the draft genome sizes of “Baizhima” and “Mishuozhima” are 267 and 254 Mb, respectively.

### Microsatellite Mining and Primer Designing

Perl scripts from MISA (Thiel et al., 2003) were used for identifying SSRs based on the reference genome sequence. Perfect microsatellites, as well as compound microsatellites interrupted by a certain number of bases were searched (Yu et al., 2016). The parameters were set for detecting mono-, di-, tri-, tetra-, penta-, and hexa-nucleotide (nt) motifs with a minimum of 10, 6, 5, 5, 5, and 5 repeats, respectively. The compound ones were defined as ≥2 repeats interrupted by ≤100 bp. Primer3 software (Untergasser et al., 2012) was employed to design up to three primer pairs to all the identified SSRs. We named all SSRs from SiSSM1 to SiSSMxx following their order on the pseudo-chromosomes and unanchored sequences. To identify the SSRs within genic regions, the general feature format (GFF) files of genes or transcripts were combined with the positions of the SSRs located on pseudo-chromosomes. The corresponding genes or transcripts linked to each SSR, along with the biological functions were retrieved from “Sinbase.” In addition, Circos (Krzywinski et al., 2009) was used to construct the diagram of the SSR density and their genomic features in sesame.

### Electronic Polymerase Chain Reaction

The primer pairs of 105,879 microsatellites located on the 13 pseudo-chromosomes were used to *in silico* amplify the genomic sequences of “Zhongzhi13,” “Mishuozhima,” and “Baizhima,” employing the software GMATA (Wang and Wang, 2016). The primer nucleotide mismatch allowed was no more than one nucleotide and other parameters were set as default. The polymorphic primers were selected based on difference in number of repeat-units present in the three genomes.

### Plant Materials and DNA Extraction

A total of 48 accessions of the cultivated sesame (*S. indicum* L., 2*n* = 26), comprising of landraces and modern cultivars grown in 12 countries of West, Central, and East Africa, were used in this study (Supplementary Table S1).

Leaves from 2 weeks old single seedling per accession were used for DNA isolation using the cetyltrimethylammonium bromide (CTAB) according to method described by Dossa et al. (2016c). DNA quality and quantity were assessed on 1.5% agarose gel and by spectrophotometry (NanoDrop 2000, Thermo Scientific, Wilmington, DE, United States), respectively. DNA samples were stored at -20°C, for further use.

### Polymerase Chain Reaction, Electrophoresis, and Data Analysis

A subset of 23 SSR markers providing coverage across all the 13 pseudo-chromosomes was selected from the entire polymorphic markers identified through electronic polymerase chain reaction (e-PCR), to validate their polymorphism potential between the 48 sesame accessions. PCR was conducted as described by Dossa et al. (2016c). Briefly, PCR was performed in a total volume of 15 μL containing 30 ng of DNA, 1 pmol of each primer, 0.2 U Taq DNA polymerase and 2× reaction mix supplied with the dNTPs and MgCl_2_. The PCR cycles were 94°C (5 min), 35 cycles of 94°C (30 s), 55°C (30 s), 72°C (30 s), followed by the extension step for 5 min at 72°C. The PCR amplicon sizes were scored in base pairs (bp) based on migration relative to the internal size standard of 400HD-ROX (Applied Biosystems, Foster, CA, United States) on an ABI 3130xl Genetic Analyzer (Applied Biosystems). Additionally, the amplified products were also electrophoretically separated on 1.5% agarose gel in TAE buffer and stained with ethidium bromide.

The number of alleles (Na), major allele frequency (MAF), and polymorphic information content (PIC) were calculated with the software PowerMarker version 3.25 (Liu and Muse, 2005). Moreover, to identify the pair-wise genetic relationships between the 48 accessions, a neighbor-joining (NJ) tree based on Nei genetic distance (Nei, 1972) was drawn in MEGA version 7 (Kumar et al., 2016).

### Development of SisatBase

The process of SisatBase development can be divided into two steps: (i) integration and consolidation of microsatellites data and (ii) developing SisatBase and embedding useful tools.

The datasets were curated to create a logic relationship among the different types of microsatellite data for their integration in SisatBase. Thereafter, SisatBase was developed using the LMAP (Linux + Apache + Mysql + Perl/PHP/Python) web application program platform. The HyperText Markup Language (HTML) and JavaScript language were also used to develop a user-friendly web interface. With the aim to enrich the functions of SisatBase, Browse, Search, customized BLAST, and MISAweb were developed for users to browse, search, and identify SSRs in the sesame genome conveniently (Altschul et al., 1997; Stein, 2013).

## Results

### Identification, Characteristics, and Genomic Distribution of SSRs in the Sesame Genome

A total of 138,194 non-redundant microsatellites were identified from 4,449 sequence scaffolds representing 94.3% of the assembled genome of sesame with an average of 507 microsatellites per Mb (**Table 1**). Mono-nucleotide and di-nucleotide SSRs were the most represented repeat types (92.5% of the whole genome SSRs) with 79% as perfect SSR types, while the remaining were in compound forms. The most prevalent motif types were A/T, accounting for 91.85% of the total mono-nucleotide repeats. For di-nucleotide motifs, the dominant motif was “AT” accounting for 50.38% of the total di-nucleotide repeats. Overall, the dominant/major motifs (A, AT, AAG/AAT, AAAT, AAAAT, and AAAAAT) were all A/T rich, whereas the absent/scarce motifs were mostly C/G rich.

**Table 1:** Characteristics of SSRs identified in the whole genome of sesame.

SSR mining		Total	
Total number of sequence scaffolds examined		4,449	
Total number of identified SSRs		138,194	
Number of sequence scaffolds containing SSR		1,279	
Number of sequence scaffolds containing more than 1 SSR		877	
Number of compound SSRs		28,666	
Number of SSRs present in genic regions		20,167	
	**Repeat type**	**Number of SSRs**	**Percentage**
	Mono-nucleotide	67,949	49.17
	Di-nucleotide	59,886	43.33
	Tri-nucleotide	9,116	6.60
	Tetra-nucleotide	933	0.68
	Penta-nucleotide	148	0.11
	Hexa-nucleotide	162	0.12
**Total**		**138,194**	**100**


**FIGURE 1:**
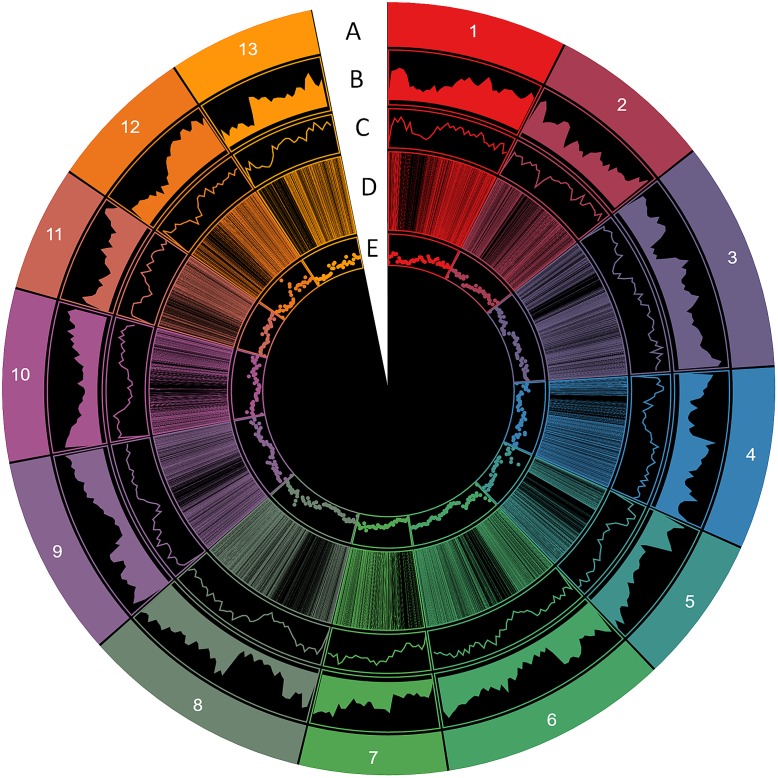
Distribution of SSR information in the sesame genome. **(A)** The 13 chromosomes, **(B)** gene density, **(C)** SSR density, **(D)** polymorphic SSRs, **(E)** genic SSRs (window of 500 kb).

**FIGURE 2:**
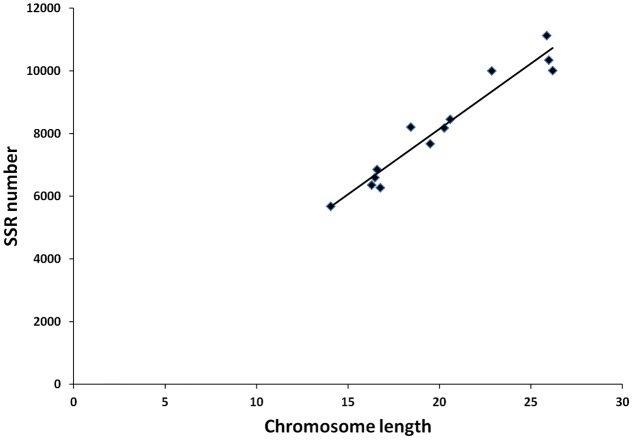
Relationships between SSR number vs chromosome length in the sesame genome.

From these microsatellites, 76.5% (105,880 SSRs) were successfully mapped onto the 13 pseudo-chromosomes (“chr”) of the sesame genome (**Table 2** and **Figure 1**). Overall, SSRs are distributed throughout the “chr” with some regions exhibiting higher density than others. The chr3 displayed the highest number of SSRs (10.5% of all mapped SSRs) followed by chr6, chr8, and chr9 accounting for 9.78, 9.46, and 9.46% of the all mapped SSRs, respectively. The chr11 harbored the lowest number of SSRs (5,686; 7.74%). Based on the physical location of each SSR and the GFF files of genes or transcripts, we uncovered that 18.84% of the total mapped SSRs were located in genic regions. Next, we estimated the relationship between the “chr” length and the number of SSRs harbored on each “chr” and found a high correlation (*r*^2^ = 0.94) (**Figure 2**).

**Table 2:** Chromosome wise distribution of SSR types in the sesame genome.

Chromosomes	Perfect types						Compound types	Total	Percent (%)
				
	Mono-	Di-	Tri-	Tetra-	Penta-	Hexa-			
chr1	3935	2498	462	57	7	8	1222	8189	7.73
chr2	4057	2438	457	45	10	13	1194	8214	7.76
chr3	5553	3174	588	72	11	16	1722	11136	10.52
chr4	4048	2469	502	55	20	7	1364	8465	7.99
chr5	3523	1945	357	37	3	9	984	6858	6.48
chr6	5164	3022	580	58	14	8	1502	10348	9.77
chr7	3157	1805	354	32	11	5	914	6278	5.93
chr8	4858	2952	589	62	11	8	1531	10011	9.46
chr9	4703	3071	644	60	7	16	1508	10009	9.45
chr10	3895	2216	400	44	5	10	1104	7674	7.25
chr11	2706	1756	309	31	7	5	872	5686	5.37
chr12	3116	1916	365	36	11	10	955	6409	6.05
chr13	3415	1798	360	45	3	5	977	6603	6.24
Total	52130	31060	5967	634	120	120	15849	105880	100.00


### Primer Designing and e-PCR Based Polymorphic Screening of the Developed SSRs among Three Sesame Genome Sequences

**FIGURE 3:**
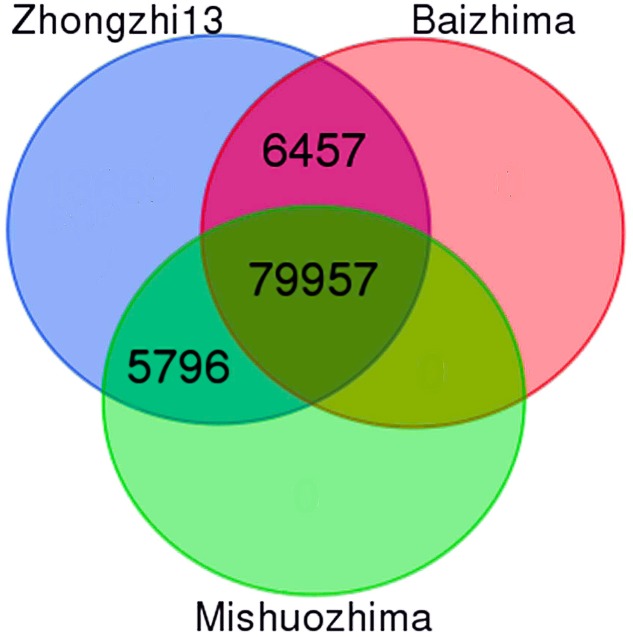
Venn diagram displaying polymorphic SSRs between three sesame genome sequences (“Zhongzhi13” as reference genome, “Mishuozhima” and “Baizhima”).

With the release of new genome sequences from two landraces (“Baizhima” and “Mishuozhima”), it is now possible to provide at the whole genome level a set of polymorphic SSRs. First, we successfully designed up to three primer pairs from flanking sequences of 104,617 SSRs (98.80% of all SSRs). Secondly, we extracted 101,930 SSRs with primers (97.4%) which were located on the 13 “chr.” Thirdly, we *in silico* amplified the three genomes mentioned above with the 101,930 SSRs. A total of 92,210 SSRs (90.5%) was conserved between the three genomes including 79.1% of total genic SSR markers. From these SSRs, 86,414 (93.7%) were polymorphic between “Zhongzhi13” and “Baizhima,” 85,753 (93%) showed polymorphism between “Zhongzhi13” and “Mishuozhima” and finally 79,957 (86.7%) SSRs were extracted as informative markers since they were polymorphic between the three genotypes (**Figure 3**). It is worthy to mention that the number of SSRs exhibiting polymorphism decreased with the increase of SSR repeat-length variation.

### Amplification and Polymorphic Potential of Selected SSRs among 48 Sesame Accessions

**FIGURE 4:**
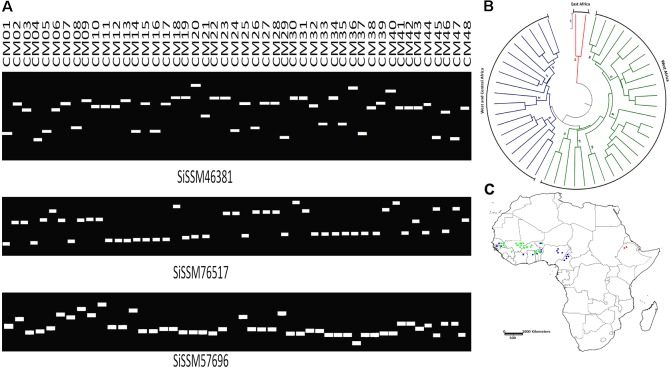
Amplification and polymorphic potential of selected SSRs among 48 sesame accessions. **(A)** Gel pictures showing polymorphic potential of some selected SSRs and allelic variations among 48 sesame genotypes. **(B)** NJ tree of the accessions used based on polymorphic SSRs. Bootstrap values ≥85 are shown. **(C)** Mapping the 48 sesame accessions based on their geographical regions. The different colors assigned represent the different clusters identified by NJ tree. Green, West Africa; blue, West and Central Africa; red, East Africa.

We selected within the 79,957 informative markers, 23 SSRs from all the 13 “chr” with the aim of confirming their allelic variation between 48 sesame genotypes. Interestingly, only two markers did not amplify three accessions probably due to DNA quality issue. More importantly, all markers (100%) were polymorphic between the 48 sesame accessions. In total, 123 distinct alleles were obtained ranging from three (SiSSM105280, SiSSM11029, SiSSM35870, SiSSM61314, SiSSM59616, SiSSM78138, SiSSM91614, and SiSSM104985) to nine alleles (SiSSM46381) with an average allele number of 4.24 per locus. The mean MAF and PIC were estimated at 0.51 and 0.60, respectively (**Figure 4A** and **Table 3**). Based on the Nei’s genetic distance between the 48 accessions, we constructed a NJ tree which divided the germplasm into three main groups (**Figure 4B**). Some geographical clustering patterns could be observed: the first group named “East Africa” gathered together the two accessions from Ethiopia. The second cluster called “West Africa” was composed of only West African accession from Senegal, Niger, Togo, Burkina Faso, Guinea, Benin, and Mali. The last group named West and Central Africa, clustered together the accessions from Nigeria, Cameroon, Senegal, Ghana, Benin, Ivory Coast, Niger, and Togo (**Figure 4C**).

**Table 3:** Polymorphism information of the 23 selected SSR markers.

Chr	SSR name	Start position (bp)	End position (bp)	Reverse primer	Forward primer	Allele number (Na)	MAF	PIC
chr1	SiSSM5580	14227382	14227544	GCTTCCACCTAGCTCGGTTAT	CCAGCAATCATGTCTGCTTAAT	4	0.61	0.6
chr1	SiSSM6522	16318566	16318595	CGTGTGCCCAATATTTGAGTT	TCAACCTCCTCCCTACACAA	4	0.74	0.6
chr2	SiSSM11029	7480687	7480700	TTGAATTTCGATCTTTCCATCA	TGGACAAAGACACAATCACACA	3	0.48	0.2
chr2	SiSSM105280	5683964	5683988	GGAGATGATTTGATTCCTTTTGA	GAAGAACAGATCGTTGGGCT	3	0.63	0.5
chr3	SiSSM22288	15034466	15034505	GCAGTGGGAGTGAGAAGAGG	TAGTGTATTCCCATCGCCCT	4	0.38	0.7
chr4	SiSSM35870	20255324	20255341	TGCATTTAAGGCTGTGCAAC	CCAGACCCAAACCCAATAGA	3	0.48	0.7
chr5	SiSSM37640	3290073	3290100	TTTGGCAAAACTGCAATGAA	CATTAACACCATTACGCAAACA	6	0.49	0.8
chr6	SiSSM46381	8881867	8881935	TGCACTGCATTGTCTCCTTT	TGCAAGGACAACCAAAATCA	9	0.45	0.8
chr7	SiSSM57696	13029902	13029961	GTCAAAATTGAGGGTTGCGT	TTCTGTCACCAAGAATTGCG	7	0.35	0.8
chr8	SiSSM61314	4072390	4072409	TTCCAATTCTACAAGCGCAG	CCGATCAAAACTAGTATGGCAA	3	0.44	0.6
chr8	SiSSM59616	391702	391727	TCATTAACCCATCATTGCGA	TGCTCACACATAACAGTTGGG	3	0.57	0.4
chr9	SiSSM76517	16006845	16006882	TCCTGAATTCAAACGCATTG	TCCTAAACCCTCTGCACCAC	8	0.59	0.7
chr9	SiSSM78138	19556070	19556099	AGCAACGATTCACGACATTG	CAACACCACCAACGCATATC	3	0.28	0.3
chr10	SiSSM84645	13921579	13921594	GATTTTGACACCTTTGCCTGA	AAAATCCTCTTTTTCCGACGA	4	0.36	0.5
chr10	SiSSM86610	18019054	18019133	ACACATACGGACAGGCACAG	ATATAGCCAGTTTGGCTGCG	4	0.47	0.7
chr11	SiSSM91614	11169519	11169554	CCAGCTCTATTGTGCGTTGA	CACTGCTTTCTCTGAAAGGCT	3	0.6	0.5
chr12	SiSSM95212	6801590	6801617	AATTGGACTCCGGCTAGGAT	CGCCCTCATCCTTACAATCT	5	0.75	0.6
chr12	SiSSM95090	6354025	6354048	AGGAAGGAGGGTGTCCCTAA	CCCCTCTCAAATAAGCCCTC	5	0.48	0.8
chr12	SiSSM97651	12374603	12374737	CGCCTTTCTCCTCCTTATCC	CATTCAGTCTTACGTCCAAATTTCT	5	0.85	0.6
chr12	SiSSM97727	12601031	12601067	ACTGCACCCTCTGCATTTTT	GCACGTGTGGGGTACCTTTA	5	0.36	0.6
chr13	SiSSM104985	14705386	14705400	GGCCAACCCTTTTCAGATTT	ATGCTCTGTGCTGATTGGTG	3	0.56	0.5
chr13	SiSSM100596	5393137	5393166	TCGAGTTGGAATGCAACAAA	CAAGTCGCCATCACACTCAT	5	0.55	0.7
chr13	SiSSM100938	6125899	6125920	TCCCAATCAGTTAGGTCGAG	TTAAGCTTAGGGGTCGGGTT	4	0.23	0.5
					**Mean**	4	0.51	0.60
					**Max**	9	0.85	0.80
					**Min**	3	0.23	0.20


### SisatBase: An Online Database for SSR Functional Analysis in Sesame

**FIGURE 5:**
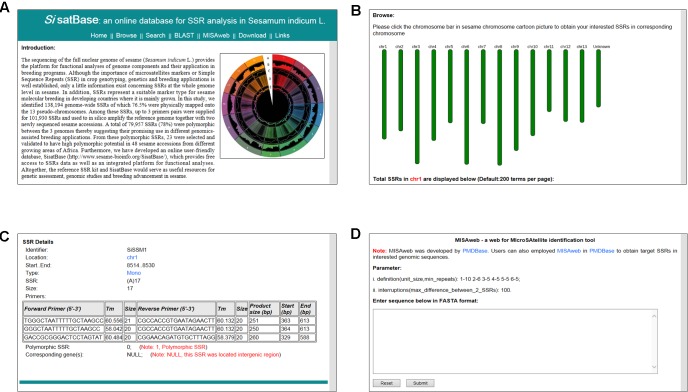
Screenshot of usage and interface of SisatBase. **(A)** Homepage of SisatBase. **(B)** Browse window displaying SSR information according to the sesame chromosome. **(C)** Details the users of SisatBase can get about each SSR. **(D)** MISAweb window to identify SSR in an interested genomic region.

In order to facilitate the exploitation of the SSRs at the whole genome level in sesame, we developed an online database with an easy-to-use interface^3^ (**Figure 5A**). SisatBase supplied basic information for SSRs, including location on chromosomes, SSR type, SSR size and up to three primer pairs for each SSR entry, as well as the functional genes associated with the SSRs. Except that, SisatBase also provided the polymorphic SSRs among different sesame genotypes. In addition, SisatBase supplied useful search tools, including keyword, SSR type, and SSR location searches, which can help users to obtain their interested SSR information (**Figures 5B,C**). Customized BLAST and MISAweb were also embedded in SisatBase to help users to get or identify conveniently SSR with primers in their interested genomic regions or genomic sequences (**Figure 5D**).

## Discussion

While the integration of molecular marker technologies have significantly improved the speed and precision of modern plant breeding, the molecular research in sesame has lagged behind other model crops mainly because sesame is a minor crop often grown by smallholders in developing countries. Hence, highly informative molecular marker systems with the advantage of easy and low-cost detection are capital for sesame breeding research. Microsatellite markers constitute undoubtedly the best candidate and in this study, we identified 138,194 SSRs at the whole genome level, along with their primer pairs and genome location.

The number of SSRs identified and the SSR density were higher than previous reports in sesame, mainly, because the genomic sequences examined in this study are more important (Wei et al., 2014; Uncu et al., 2015; Dossa, 2016). Furthermore, by exploiting the latest version of the reference genome, we are able to provide the accurate position of SSRs in the sesame genome compared with previous reports. This would be helpful for gene fine-mapping and association analysis in sesame. Mono-nucleotide and di-nucleotide repeats accounted for 92.5% of the whole genome SSRs in sesame. Our results are in agreement with conclusions of Cardle et al. (2000) and Sonah et al. (2011), who identified mono-nucleotide and di-nucleotide repeats as the predominant repeat types in several plant genomes including *Arabidopsis thaliana*, *Brachypodium distachyon*, *Sorghum bicolor*, *Oryza sativa*, *Medicago truncatula*, and *Populus trichocarpa*. Similarly to previous reports of Wei et al. (2014), Uncu et al. (2015), and Dossa (2016), the distribution of A/T rich motif as the major motif is highly in accordance with the AT (0.68%) vs GC (0.32%) content in the sesame genome (Wang et al., 2014a). The same findings were also observed in *Brassica rapa* (Xu et al., 2010; Shi et al., 2014), *Brassica napus* (Cheng et al., 2009), *Brassica oleracea* (Li et al., 2011), cucumber (Cavagnaro et al., 2010). The high correlation of SSR number and pseudo-chromosome length suggested that this type of DNA considerably increase the length of the sesame pseudo-chromosomes.

In sesame, SSRs were more concentrated in the intergenic regions compared to genic regions which is consistent with findings in *Sativa japonica* (Zhang et al., 2007), maize (Xu et al., 2013), and other crops (Hancock, 1995). The landraces “Baizhima” and “Mishuozhima” exhibited similar polymorphic rates with the genome of “Zhongzhi13.” This suggested that the two landraces are much closer to each other than the elite variety “Zhongzhi13.” Our findings are in agreement with the conclusions of Wei et al. (2016) who found that the two landraces clustered together and were more closely related in the phylogenetic tree compared to “Zhongzhi13.” We further discovered that the majority of genic SSRs in the sesame genome have been found within the conserved markers between the three genotypes. This result is understandable given that SSRs within genic regions are associated with genes which constitute the genome component more conserved within species (Xiao et al., 2016). On the other hand, this implies that the conserved set of SSRs might be related to important genes which were retained during improvement from landraces to elite cultivar, as demonstrated in soybean (Zhou et al., 2015). Therefore, we infer that these genic informative microsatellites may be linked to some important biological functions and could be potential tools for sesame breeding (Lata et al., 2014; Dossa et al., 2016a,b).

In our knowledge, there are no specific molecular markers developed for other related species in the *Sesamum* genus. It has been demonstrated that SSR markers have a good transferability between species of the same genus or even in the same taxa (Fan et al., 2013; Buso et al., 2016; Huang et al., 2016; Thakur et al., 2017). In sesame, Uncu et al. (2015) uncovered a high rate of SSR marker transferability between the cultivated species *S. indicum* and the proposed wild ancestor species *S. malabaricum*. In addition, different sets of SSR markers developed in the cultivated sesame also yielded good amplicons in the wild-related species including *Sesamum radiatum*, *S. angustifolium*, *S. latifolium*, *S. angolense* (Zhang H. et al., 2012; Nyongesa et al., 2013; Wu et al., 2014). Based on these reports, we speculate that our developed informative SSR markers might be relevant for other wild-related species of the *Sesamum* genus. This will be significant for the genetic improvement of the cultivated form by exploiting the potential of the wild-related species (Dossa et al., 2017). Such transferable SSR markers between *Sesamum*-related species could be used for conducting macro-synteny studies, genetic mapping, and molecular breeding. Therefore, in future studies, we will employ several wild-related species of the *Sesamum* genus as well as a diverse panel of the cultivated sesame to evaluate the cross-species transferability of our developed SSR markers and initiate genetic researches in the wild-related species of the *Sesamum* genus.

Although some SSR sets have been previously identified in the sesame genome, transcriptome, etc. (Spandana et al., 2012; Zhang H. et al., 2012; Wei et al., 2014; Uncu et al., 2015; Dossa, 2016), information regarding their amplification efficiency and polymorphic potential is limited. In the present study, we took advantage of the three available sequenced genomes to screen for amplification efficiency and polymorphism potential of our developed SSR markers. This led to the identification of 79,957 informative SSR markers of which 23 selected SSRs successfully discriminated 48 genotypes from Africa based on their geographical origins. This result suggested that e-PCR is a useful strategy for a rapid screening and an effective identification of informative markers (Wang and Wang, 2016; Xiao et al., 2016). In the works of Dossa et al. (2016c), 33 polymorphic SSRs were employed to assess the genetic diversity of 96 sesame accessions from Africa and Asia which resulted in a high genetic diversity within the African germplasm. The 23 selected SSRs used in the present study to scan the diversity of 48 African accessions were all polymorphic and yielded comparable alleles number (123 vs 137) although fewer genotypes were examined here. Similarly, a high genetic diversity was also observed in the studied germplasm proving that the global 79,957 informative SSR markers could be effectively considered as the reference SSR for large-scale genotyping and molecular breeding research in sesame (Billot et al., 2012).

All SSR data were integrated into SisatBase which also supplied useful and user-friendly tools to assist users to extract more information related to SSR markers in the sesame genome. The database will be continuously updated with new versions of the sesame genome. Moreover, with the aim of extending the utility of SisatBase over other species of the *Sesamum* genus, new information about the cross-species transferable SSR markers as well as novel and specific SSRs for each species will be supplied in the future.

## Conclusion

In conclusion, based on the latest version of the sesame reference genome and the two newly released genome sequences, we identified 138,194 SSRs of which 79,957 are proposed as the reference SSR for future genetics/genomics and breeding studies in sesame. All microsatellite data reported in this study are integrated into a user-friendly online database (SisatBase) for a convenient exploitation and further functional analyses. These tools will undoubtedly help to speed-up sesame molecular breeding especially in the developing countries.

## Author Contributions

KD and JY produced the sesame SSR data, developed the online database, and drafted the manuscript. KD performed the experiments. BL, NC, and XZ designed the project, supervised the works, and revised the draft manuscript. All authors have read and approved the final manuscript.

## Conflict of Interest Statement

The authors declare that the research was conducted in the absence of any commercial or financial relationships that could be construed as a potential conflict of interest.

## Footnotes

1 www.ocri-genomics.org/Sinbase/index.html

2 http://ncgr.ac.cn/SesameFG/

3 http://www.sesame-bioinfo.org/SisatBase/
